# Intra-articular injections of high-molecular-weight hyaluronic acid have biphasic effects on joint inflammation and destruction in rat antigen-induced arthritis

**DOI:** 10.1186/ar1725

**Published:** 2005-03-31

**Authors:** Andreas Roth, Jürgen Mollenhauer, Andreas Wagner, Reneè Fuhrmann, Albrecht Straub, Rudolf A Venbrocks, Peter Petrow, Rolf Bräuer, Harald Schubert, Jörg Ozegowski, Gundela Peschel, Peter J Müller, Raimund W Kinne

**Affiliations:** 1 Department of Orthopaedics, 'Rudolf-Elle' Hospital, Friedrich Schiller University Jena, Eisenberg, Germany; 2 Department of Biochemistry, Rush Medical College Head, Chicago, Illinois, USA; 3 Institute of Pathology, Friedrich Schiller University Jena, Germany; 4 Institute of Animal Studies, Friedrich Schiller University Jena, Germany; 5 Institute of Biochemistry 2, Friedrich Schiller University Jena, Germany; 6 Hans Knoell Institute for Natural Products Research, Jena, Germany; 7 Experimental Rheumatology Unit, Friedrich Schiller University Jena, Germany

## Abstract

To assess the potential use of hyaluronic acid (HA) as adjuvant therapy in rheumatoid arthritis, the anti-inflammatory and chondroprotective effects of HA were analysed in experimental rat antigen-induced arthritis (AIA). Lewis rats with AIA were subjected to short-term (days 1 and 8, *n *= 10) or long-term (days 1, 8, 15 and 22, *n *= 10) intra-articular treatment with microbially manufactured, high-molecular-weight HA (molecular weight, 1.7 × 10^6 ^Da; 0.5 mg/dose). In both tests, 10 buffer-treated AIA rats served as arthritic controls and six healthy animals served as normal controls. Arthritis was monitored by weekly assessment of joint swelling and histological evaluation in the short-term test (day 8) and in the long-term test (day 29). Safranin O staining was employed to detect proteoglycan loss from the epiphyseal growth plate and the articular cartilage of the arthritic knee joint. Serum levels of IL-6, tumour necrosis factor alpha and glycosaminoglycans were measured by ELISA/kit systems (days 8 and 29). HA treatment did not significantly influence AIA in the short-term test (days 1 and 8) but did suppress early chronic AIA (day 15, *P *< 0.05); however, HA treatment tended to aggravate chronic AIA in the long-term test (day 29). HA completely prevented proteoglycan loss from the epiphyseal growth plate and articular cartilage on day 8, but induced proteoglycan loss from the epiphyseal growth plate on day 29. Similarly, HA inhibited the histological signs of acute inflammation and cartilage damage in the short-term test, but augmented acute and chronic inflammation as well as cartilage damage in the long-term test. Serum levels of IL-6, tumour necrosis factor alpha, and glycosaminoglycans were not influenced by HA. Local therapeutic effects of HA in AIA are clearly biphasic, with inhibition of inflammation and cartilage damage in the early chronic phase but with promotion of joint swelling, inflammation and cartilage damage in the late chronic phase.

## Introduction

Rheumatoid arthritis (RA), a chronic systemic disease primarily affecting the joints, is characterised by progressive destruction of cartilage and bony structures of the joints [1,2]. Its social impact results from the personal suffering of patients as well as from medical and indirect costs [3].

Hyaluronic acid (HA) is a large linear glycosaminoglycan composed of repeating disaccharide units of glucuronic acid and *N*-acetylglucosamine, linked via the 1–4 position of the sugar rings [4]. The synovial fluid in the joint consists of ultrafiltrated plasma and HA, the latter being produced by type-B synoviocytes of the lining layer [5]. Inflammatory changes lead to depolymerisation of HA, resulting in a decrease of its molecular weight and its concentration [6]. Its lubricant properties decrease, contributing to the destruction of cartilage and bone [7].

HA protects cells and anatomical structures against mechanical overloading due to its viscoelastic characteristics [8]. The viscosity of the synovial fluid is reduced in patients with RA [9], a deficit that can be balanced by the supply of exogenous HA [10]. In addition, the production of endogenous synovial HA is stimulated via the supply of exogenous HA [11].

RA is characterised by a loss of proteoglycans in the affected joints [12,13]. HA possesses chondroprotective effects [10,14] and is reported to inhibit the loss of proteoglycans from the matrix of joint cartilage [15,16]. HA also blocks the loss of proteoglycans caused by the addition of catalytic cytokines to cultivated cartilage [17,18] and suppresses the degradation of cartilage matrix mediated by fibronectin fragments [19,20]. HA is also reported to protect the cartilage against proteoglycan loss, against chondrocyte cell death caused by free oxygen radicals, IL-1, or mononuclear-cell-enriched medium, and against other alterations [14,15,21-24]. Cartilage degradation induced by neutrophil leukocytes is also reduced by HA *in vitro *[25]. Injection of exogenous HA induces a decrease of inflammatory and proliferative processes within the synovium [26]. Also, HA inhibits the proliferation [27] and migration of white blood cells [28], and affects their adherence, chemotaxis, and phagocytosis properties [11,29,30]. Degradation of HA by reactive oxygen species, on the other hand, may reduce the protective properties of HA [14,31].

In spite of the known potential benefits of HA on a number of pathological features of RA, a general estimate of its validity for the treatment of RA is still lacking, particularly in terms of experimental studies in animal models of arthritis. The present study was therefore designed to examine the effects of HA in rat antigen-induced arthritis (AIA). This experimental monoarticular arthritis shares some characteristics of RA; for example, hyperplasia of the synovial membrane, inflammatory infiltration of the joints, and destruction of cartilage [32]. This model is also useful to characterise treatment responses; for example, the reduction of inflammation or changes in the synovial connective tissue [33].

## Materials and methods

### Animals

Female Lewis rats (10–12 weeks of age) were obtained from the Institute of Animal Studies, Friedrich Schiller University Jena, Germany. The rats were housed under standard conditions, in a 12-hour light/dark cycle. The animals were fed with standard rodent chow and water *ad libitum*. The rats were divided into two groups: non-arthritic animals (*n *= 6) and arthritic animals (*n *= 40). The latter were subdivided into the following groups (each *n *= 10): untreated AIA rats, short-term test (US); untreated AIA rats, long-term test (UL); HA-treated AIA rats, short-term test (HS); and HA-treated AIA rats, long-term test (HL). All animal studies were approved by the governmental committee for animal protection.

### Hyaluronic acid

Pyrogen-free, sterile-filtered HA with a molecular weight of 1.7 × 10^6 ^Da was used, obtained by biotechnological fermentation from *Streptococcus equisimilis ssp. zooepidemicus V 2541*. This bacterial HA, also called non-animal-source hyaluronan, is completely identical to human HA. The content of pyrogen was minimised to less than 0.05 IE/ml HA by cleaning steps, therefore fulfilling the demands of the *European Pharmacopeia *(Supplement 2001, page 1472). The zero-viscosity of the purified 1.0% high-molecular-weight HA (molecular weight, 1.7 × 10^6 ^Da) in 0.9% NaCl solution amounted to h0 = 10.74 Pa s. The injection units contained 10 mg HA in 1 ml of 0.9% NaCl.

### Induction of AIA

All experimental animals were immunised by two subcutaneous injections (days -21 and -14) of 0.5 g methylated bovine serum albumin (mBSA), dissolved in 0.5 ml saline and emulsified with 0.5 ml complete Freund's adjuvant [32,34]. Knee monoarticular arthritis was induced 2 weeks after the second immunisation via a single joint injection of 0.5 mg mBSA (50 μl of 10 mg/ml mBSA dissolved in 0.9% NaCl) into the right knee joint (day 0 of AIA). The left knee remained without injection.

### Treatment with HA

On day 1 of AIA, all 40 arthritic animals received an intra-articular injection into the right inflamed knee joint. HA-treated AIA rats (groups HS and HL) received in each case 0.5 mg HA (50 μl of 10 mg/ml HA in 0.9% NaCl), whereas the untreated AIA rats (groups US and UL) received 50 μl PBS. The AIA rats of the long-term test received further injections at the beginning of each subsequent week (days 8, 15, and 22): the HL group received 50 μl HA, and the UL group received 50 μl PBS.

The short-term test (groups US and HS) was terminated 1 week after the first injection of HA or PBS (day 8). The long-term test (groups UL and HL) was terminated 1 week after the fourth injection (day 29).

In all cases, the contralateral (left) knee joint remained untreated. The group of six non-arthritic animals without AIA (12 weeks of age) served for the collection of normal values.

All injections (including those necessary to induce immunisation and knee AIA) were performed under ether anaesthesia. At the end of the experiment, the animals were sacrificed using an overdose of CO_2 _and cervical dislocation.

### Collection of samples

Blood samples were collected by heart puncture after opening the thorax. The blood was centrifuged for 10 min at 3000 × *g *and ambient temperature. The serum was divided into three portions of at least 250 μl and was frozen at -80°C until analysis.

The knee joints were disconnected from the long bones and stored in 6% formaldehyde. In order to ensure an optimal impregnation with formaldehyde, the adhering remainders of the long bones were kept very short (approximately 1.0 cm above and below the joint space) and the dorsal joint capsule was opened.

### Evaluation of arthritis

Joint swelling, body weight, and the general state of the animals were regularly monitored. The measurements of weight and mediolateral joint diameter took place on days 0, 1, 4, 8, 15, 22, and 29. The mediolateral joint diameter was measured using a vernier caliper [32,34].

### Histological analyses

All preparations were stored in 6% formaldehyde for 24 hours. Decalcification in ethylenediamine tetraacetic acid subsequently took place and the preparations were embedded in paraffin. After the removal of paraffin, 5-μm thick sections were cut [35].

For the assessment of the histological arthritis scores, the sections were stained with haematoxylin and eosin. All slides were evaluated by an independent observer who was blinded to the design and details of the study. In all cases, three sections per knee joint were examined and scored using a semiquantitative scale.

The extent of acute joint inflammation – as defined by the degree of infiltration of the synovial membrane by polymorphonuclear leukocytes, and defined by the exudation of granulocytes in the joint space – was evaluated in each case with 0 = no changes, 1 = mild changes, 2 = moderate changes, and 3 = severe changes. In addition, the presence (score 1) or absence (score 0) of fibrin exudation in the joint space and periarticular inflammation was assessed, resulting in a maximum total score of 8 for acute inflammation.

Chronic joint inflammation – based on the parameters hyperplasia of synovial lining cells, infiltration by mononuclear cells, and fibrosis of synovial membrane or periarticular tissue – was evaluated with a score of 0–3, resulting in a maximum total score of 9.

The extent of the damage to articular cartilage and adjacent bone structures (cell necrosis, structural bone, and cartilage defects) was evaluated with score 0 = no damage, score 1 = <5% of the cartilage surface affected, score 2 = 5–10% of the cartilage surface affected, score 3 = 10–50% of the cartilage surface affected, and score 4 = >50% of the cartilage surface affected (maximal total score of 4).

Safranin O staining was performed to estimate the proteoglycan content in the cartilage [36-38]. In order to obtain comparable histological results, all slides were stained using exactly the same procedure [39]. The preparations were analysed under defined conditions using a Zeiss microscope Axiovert 200 M (20 × magnification) (Carl Zeiss, Göttingen, Germany)] and the results were stored as pixel pictures. The staining intensity was determined in 175 × 25 mm^2 ^areas, using Scion Image software (Scion Corporation, Frederick, MD, USA).

**Figure 1:**
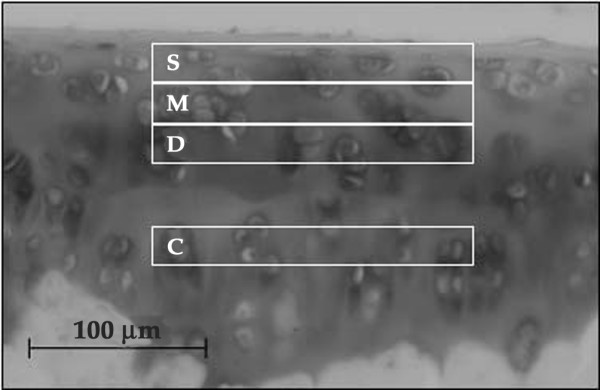
Measurement frames for Safranin O staining of the knee joint cartilage. After elimination of green tones and transformation of all red tones into grey tones, the staining intensity (a measure of the proteoglycan content) was determined in the following layers: S, superficial layer; M, middle layer; D, deep layer; and C, calcified cartilage.

First, the staining intensity (red) at the epiphyseal growth plate of the femoral condyle of non-arthritic and arthritic animals was measured (maximum value 255). The arithmetic mean obtained from these values was used as a reference value (232 [= 100%]). The measurements of articular cartilage took place at the most distal point of the curvature of the femoral condyle. In each case, values were obtained for the superficial layer, middle layer, and deep layer of the hyaline cartilage, as well as for the calcified cartilage layer (Fig. 1). Data were expressed as a percentage of the reference value. Subsequently, the values of the contralateral, non-arthritic knee joint (left) were subtracted from the arthritic knee (right), resulting in negative values in the case of proteoglycan loss.

### Cytokine and serum glycosaminoglycan evaluation

The serum levels of IL-6, tumour necrosis factor alpha (TNF-α) and glycosaminoglycan (GAG) were determined at the end point of the short-term test (day 8) and at the endpoint of the long-term test (day 29).

The serum levels of IL-6 and TNF-α were determined using a commercial sandwich ELISA kits for rats according to the manufacturer's instructions (Biosource International, Camalliro, CA, USA). The detection limits were 8 pg/ml for IL-6 and 4 pg/ml for TNF-α. According to the manufacturer, there was no cross-reactivity with other rat cytokines.

The serum levels of total GAG were measured in non-diluted serum with a commercially available kit. The standard values for healthy rats were 10.8–17.4 mg/l (Glycane T Labor + Diagnostica, Freital, Germany).

### Statistics

Statistical evaluations were carried out using the programme SigmaStat 2.0. Since nearly all data were not normally distributed, the non-parametric Mann–Whitney U test was used. Data were expressed as means and standard errors of the means. *P *≤ 0.05 was considered statistically significant for α.

In cases in which *P *values for α were at the limits of significance (0.05 ≤ *P *≤ 0.1; joint swelling day 29, cartilage damage day 8), the statistical power of the U test was determined using the actual difference at a given time point as delta.

Because for the time period from day 0 to day 8 the procedure and results did not differ between the US and UL groups or between the HS and HL groups, respectively, the values from the short-term test and the long-term test were pooled for statistical evaluation of this period in both cases.

## Results

### Body weight

At baseline, the body weight was 188 ± 29 g (untreated AIA rats) and 197 ± 22 g (HA-treated AIA rats). After a plateau between day 0 and day 8 in both untreated rats and HA-treated AIA rats, the body weight rose in concomitance with the decrease of arthritis severity. At the end of the long-term test (day 29), the animals weighed 213 ± 15 g (untreated AIA rats) and 230 ± 13 g (HA-treated AIA rats). The differences between the groups did not reach statistical significance at any time point.

### Joint swelling

**Figure 2:**
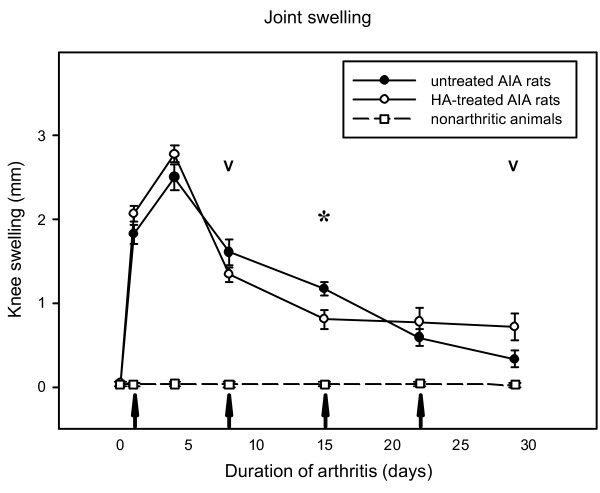
Time course of knee joint swelling. Joint swelling (difference between the bilateral diameter of the right knee and the left knee) in untreated antigen-induced arthritis (AIA) rats and in hyaluronic acid (HA)-treated AIA rats. V, end of the short-term test (day 8) and end of the long-term test (day 29). The arrows indicate the days of intra-articular injection of HA (days 1, 8, 15, and 22). In the short-term test there was no significant difference between HA-treated rats and untreated AIA rats. In the long-term test HA-treated AIA rats showed significantly reduced values on day 15 (* *P *< 0.05). On day 29 there were no longer differences between the two groups; if at all, the swelling in the HA-treated group was somewhat higher than in untreated AIA group.

On day 1, AIA developed as a significant swelling of the right knee joint in all animals (Fig. 2). The swelling increased up to day 4 in untreated AIA rats (*P *< 0.001, *n *= 20), significantly decreasing on day 8 (*P *< 0.001, *n *= 20). The swelling then continued to slowly decrease until day 29 (*P *< 0.001, *n *= 10). At all time points after initiation of AIA, the swelling remained significantly higher compared with the baseline levels on day 0 (Fig. 2).

Intra-articular treatment with HA did not significantly affect the degree of joint swelling on days 1, 4, and 8 (Fig. 2). On day 15 (groups UL and HL, *n *= 10 each) there was a significant reduction of joint swelling in the HA-treated AIA group compared with the untreated AIA group (*P *< 0.05). On day 22 the swelling was no longer significantly different from the untreated AIA group (*P *= 0.37); in fact, it was even somewhat higher. On day 29 (end of the long-term test) the small increase of joint swelling in the HA-treated AIA group persisted (as compared with the untreated AIA group), although without reaching statistical significance (power 1 β = 0.851).

In general, therefore, HA seemed to positively affect the early chronic phase of AIA (day 15), but did not have an influence on the acute or late chronic phases of AIA, at least in terms of joint swelling.

### Loss of proteoglycans from the epiphyseal growth plate of the femoral condyle and the articular cartilage

**Figure 3:**
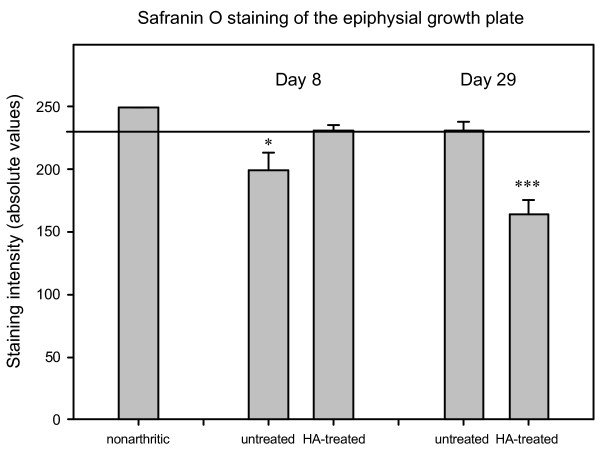
Safranin O staining intensity in the epiphyseal growth plate of the femoral condyle. The reference value of 232 (100%; continuous line) was obtained by computing all available values from both non-arthritic rats and antigen-induced arthritis (AIA) rats. In untreated AIA rats, the right (arthritic) joint showed a significant reduction of proteoglycan content of the epiphysis in the short-term test (day 8; *P < 0.05). This loss was not observed following hyaluronic acid (HA) treatment (day 8). The latter values were comparable with non-arthritic animals and with the contralateral joint (data not shown). Long-term treatment with HA (day 29) induced a significant loss of proteoglycans in the epiphyseal growth plate (*** *P *< 0.001). In contrast, the arthritic joints of untreated AIA rats showed values comparable with non-arthritic rats and contralateral joints (not shown).

In the short-term test (day 8), the untreated AIA group was characterised by a significant decrease of the proteoglycan content in the epiphyseal growth plate of the arthritic right knee compared with the contralateral knee or with the right knee joint of non-arthritic animals (in both cases, *P *< 0.05; Fig. 3). Treatment with HA prevented this loss, maintaining proteoglycan levels close to those of non-arthritic animals.

**Figure 4:**
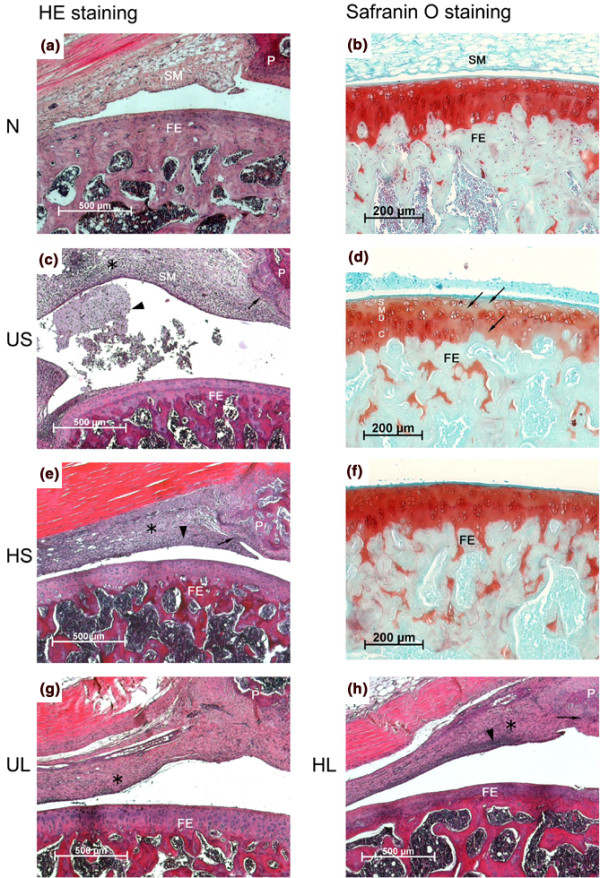
Histological findings in synovial tissue and articular cartilage: haematoxylin and eosin (HE) staining (**a**, **c**, **e**, **g**, and **h**) for acute inflammation (arrowheads), chronic inflammation (*), and cartilage damage (arrows), as well as Safranin O staining (**b**, **d**, and **f**) for proteoglycan depletion (arrows). Images are shown for non-arthritic rats (a and b), untreated antigen-induced arthritis (AIA) rats (day 8, c and d; day 29, g), and hyaluronic acid hyaluronic acid (HA)-treated AIA rats (day 8, e and f; day 29, h). The bar indicates the distance in the histological section. SM, synovial membrane; P, patella; FE, femur. Safranin O staining: S, superficial layer; M, middle layer; D, deep layer; and C, calcified cartilage. N, non-arthritic rats; US, untreated AIA, short-term test; HS, HA-treated AIA, short-term test; UL, untreated AIA, long-term test; HL, HA-treated AIA, long-term test.

**Figure 5:**
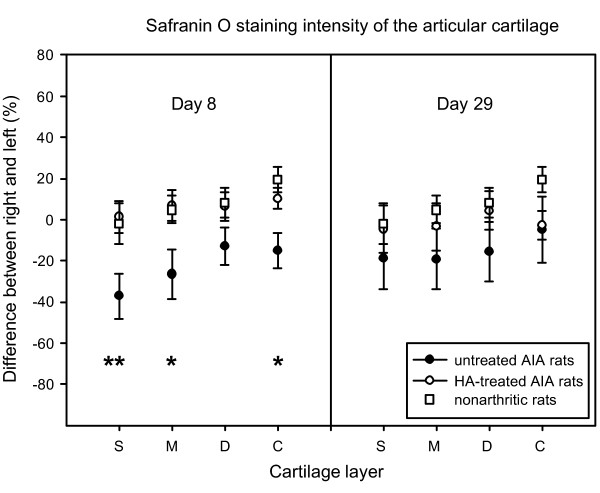
Safranin O staining intensity in different layers of the articular cartilage. Comparisons were made for different layers (S, superficial layer; M, middle layer; D, deep layer; and C, calcified cartilage) between non-arthritic rats, untreated antigen-induced arthritis (AIA) rats, and hyaluronic acid (HA)-treated AIA rats in terms of relative differences between right (arthritic) and left (contralateral) joints. In the short-term test (day 8) untreated AIA rats showed a reduced proteoglycan content in the superficial layer (** *P *= 0.01), middle layer (* *P *< 0.05), and calcified cartilage (* *P *< 0.05). HA-treated AIA rats showed proteoglycan contents comparable with those of non-arthritic rats in all layers. In the long-term test (day 29), untreated AIA rats also showed reduced proteoglycan contents in all layers compared with non-arthritic rats, but no statistical significance was attained. HA-treated AIA rats showed proteoglycan contents comparable with those of non-arthritic rats.

In terms of individual zones of the articular cartilage, the untreated AIA rats underwent a change of -37% in the superficial layer, -26% in the middle layer, -13% in the deep layer, and -15% in the calcified cartilage layer (Figs 4b,d and 5). At this time point, treatment with HA was significantly effective in preventing the proteoglycan loss in the superficial layer (*P *< 0.01), the middle layer (*P *< 0.05), and the calcified cartilage layer (*P *< 0.05; Figs 4b,f and 5). In all layers, the proteoglycan content reached normal levels.

In the long-term test (day 29) there was no significant loss of proteoglycan content in the epiphyseal growth plate of untreated AIA rats (see Fig. 3). However, treatment with HA was characterised by a significant proteoglycan loss in the growth plate of the arthritic right knee compared with the contralateral knee or with the right knee joint of non-arthritic animals (*P *< 0.001; Fig. 3).

In the different layers of the articular cartilage, the untreated AIA rats no longer showed any significant proteoglycan loss; that is, there were no significant differences between the right knee joints and left knee joints of AIA rats, or between the right knee joint of AIA rats and the right knee joint of non-arthritic animals (Fig. 5; Safranin O staining data not shown). Treatment with HA did not significantly affect the proteoglycan content in any layer of the articular cartilage.

### Histological scores of arthritis and cartilage damage

**Figure 6:**
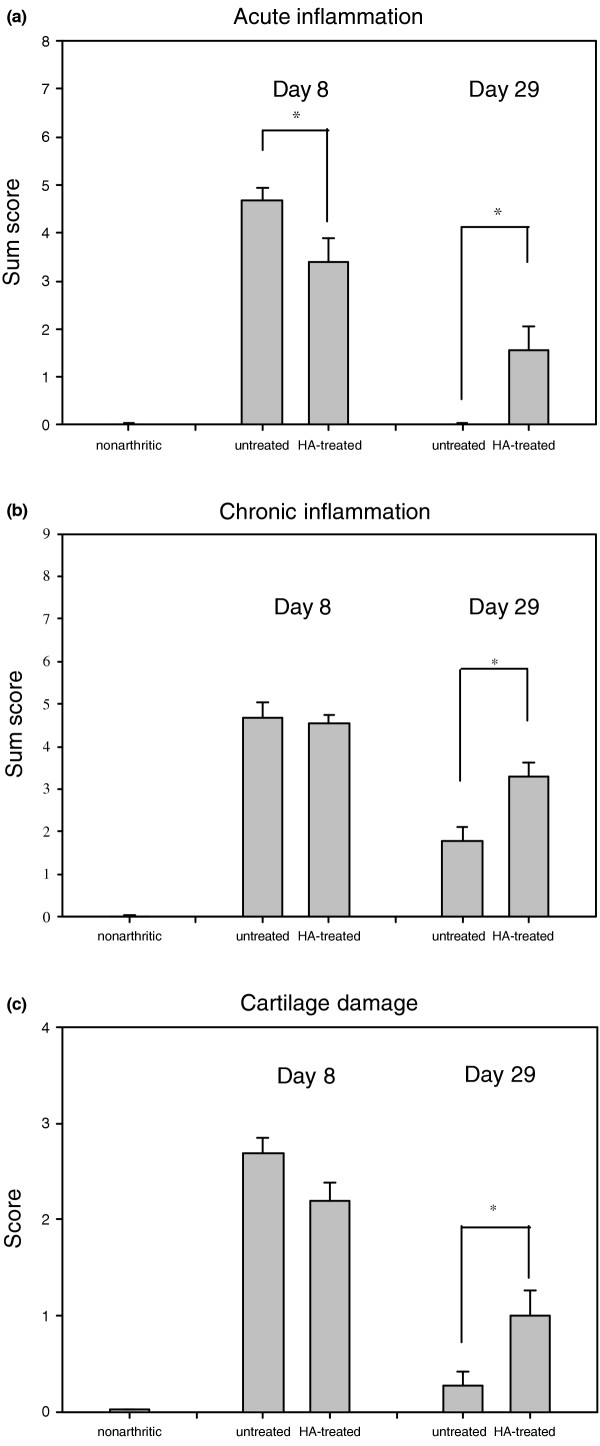
Histological scores. In the short-term test, hyaluronic acid (HA)-treated antigen-induced arthritis (AIA) rats showed a significant reduction of **(a) **the acute inflammation score compared with untreated AIA rats (* *P *< 0.05). The score of **(b) **chronic inflammation and **(c) **cartilage damage did not show significant differences between HA-treated rats and untreated AIA rats. In the long-term test, (a) the acute inflammation was reduced in both AIA groups compared with that in the short-term test (HA-treated AIA rats, *P *< 0.05; untreated AIA rats, *P *< 0.001; significance not indicated); nonetheless, the HA-treated AIA rats showed significantly higher scores than untreated AIA rats on day 29 (* *P *< 0.05). (b) The scores of chronic inflammation were reduced in both AIA groups compared with the short-term test (untreated AIA rats, *P *< 0.001; HA-treated AIA rats, *P *< 0.01; significance not indicated); nonetheless, the HA-treated rats showed significantly higher scores than untreated AIA rats on day 29 (* *P *< 0.05). (c) The cartilage damage was relatively low in both untreated rats and HA-treated AIA rats, but HA-treated AIA rats showed a significantly higher damage (* *P *< 0.05).

In the short-term test (day 8) there was a strong acute inflammation in untreated AIA rats (Fig. 6a). Treatment with HA significantly reduced the acute inflammation compared with the untreated AIA group (*P *< 0.05; Figs 4a,c,e and 6a). Notably, the untreated AIA group underwent a complete, spontaneous remission of the acute inflammation score from day 8 to day 29 (acute inflammation score nearly 0 in the long-term test; Figs 4c,g and 6a). The HA-treated AIA group in the long-term test clearly improved compared with the short-term test (*P *< 0.05), but it still showed significantly higher, residual acute inflammation than the untreated AIA group (*P *< 0.05; Figs 4e,g,h and 6a).

In the short-term test (day 8) a clear score for chronic inflammation was also observed (Figs 4a,c,e and 6b), without significant differences between untreated and HA-treated AIA groups. The chronic inflammation significantly decreased from day 8 to day 29 in untreated and HA-treated AIA (group US versus group UL, *P *< 0.001; group HS versus group HL, *P *< 0.01; Figs 4c,e,g,h and 6b). Unexpectedly, however, on day 29 the chronic inflammation score was more pronounced in the animals treated with HA compared with the untreated AIA group (*P *< 0.05; Figs 4g,h and 6b).

In terms of cartilage damage, the untreated AIA group was characterised by a maximum individual score of 3; that is, the maximal possible score of 4 was not observed (Fig. 6c). On day 8, the mean cartilage damage was somewhat more pronounced in the untreated AIA group, but without significant differences in comparison with the HA-treated AIA group (power 1 - β = 0.821). From day 8 to day 29, the cartilage damage decreased significantly in untreated rats and HA-treated AIA rats (group US versus group UL, *P *< 0.001; group HS versus group HL, *P *< 0.01). In the long-term test (day 29), however, the cartilage damage was significantly higher in the animals treated with HA than in the untreated AIA group (*P *< 0.05; Fig. 6c).

### Systemic cytokine levels

**Figure 7:**
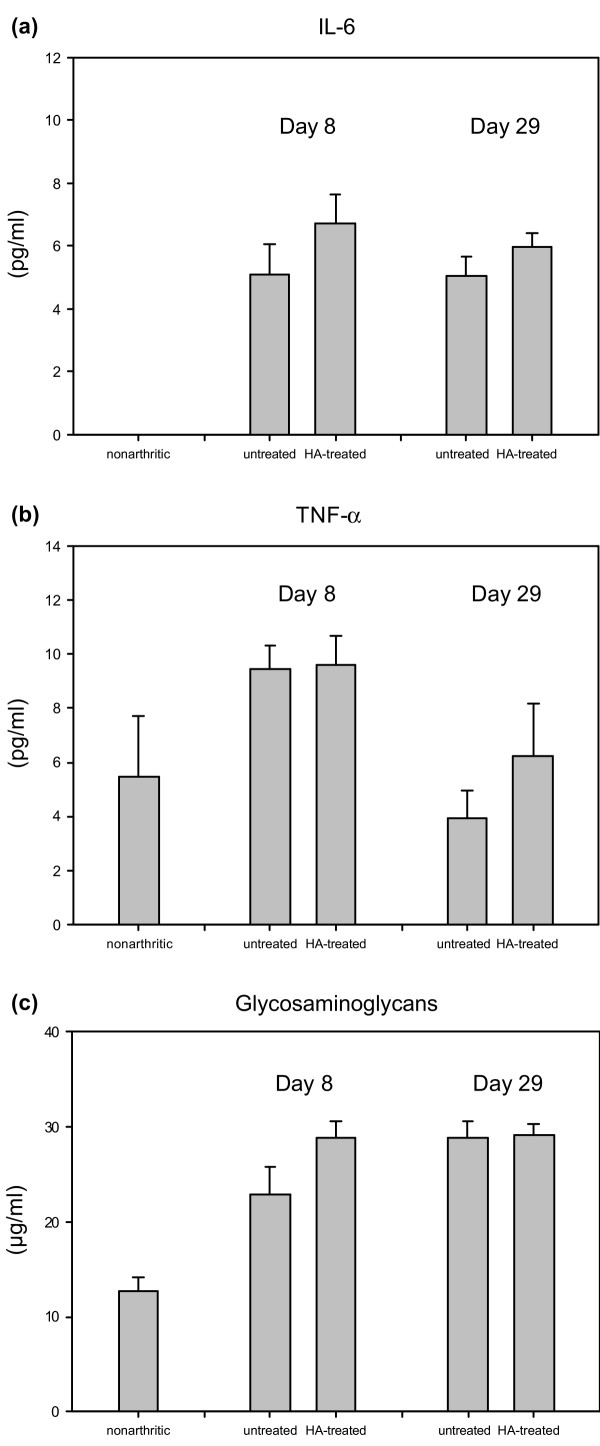
Serum levels of IL-6, tumour necrosis factor alpha (TNF-α) and glycosaminoglycan. During the course of antigen-induced arthritis (AIA), **(a) **IL-6 levels in non-arthritic rats showed values below the detection limit of the assay. Untreated and hyaluronic acid (HA)-treated AIA rats showed significantly increased values in the short-term test and in the long-term test compared with non-arthritic rats (all *P *< 0.001; data not shown), but HA treatment resulted in IL-6 levels comparable with those of untreated AIA. **(b) **Regarding TNF-α levels, the only change was a short-term, non-significant increase in all AIA rats, whether HA-treated or untreated. **(c) **Serum values of glycosaminoglycan increased significantly in all AIA rats compared with non-arthritic animals (non-arthritic animals versus untreated AIA rats [short-term test], *P *< 0.05, non-arthritic animals versus HA-treated AIA rats, untreated AIA rats [long-term test] and HA-treated AIA rats [long-term test], *P *< 0.001; significance not indicated). There were no differences between short-term tests and long-term tests, or between HA-treated rats and untreated AIA rats.

In non-arthritic animals, the serum IL-6 levels were below the detection limit of the assay (Fig. 7a). Untreated AIA rats had significantly elevated IL-6 levels both in the short-term test and in the long-term test (*P *< 0.001 in both cases; significance not indicated in Fig. 7). Treatment with HA did not significantly influence IL-6 levels at either time point (Fig. 7a).

As for TNF-α, non-arthritic animals had mean serum levels of 5.45 ± 4.56 pg/ml (Fig. 7b). In the short-term test these values were increased both in the untreated and in the HA-treated AIA groups, but not to a significant degree. In the long-term test, the mean TNF-α levels were very similar to those of non-arthritic animals. Treatment with HA did not significantly influence TNF-α levels at either time point (Fig. 7b).

### Serum GAG levels

In non-arthritic animals, the mean serum levels of GAG were 12.70 ± 3.30 μg/ml (Fig. 7c). Untreated AIA rats had significantly higher GAG levels than non-arthritic animals in the short-term test and in the long-term test (*P *< 0.05 and *P *< 0.001, respectively; significance not indicated in Fig. 7). Treatment with HA had no influence on this parameter at either time point (Fig. 7c).

## Discussion

### Clinical parameters of arthritis

The time course of AIA was similar to that described by other authors [32,34], confirming that the present results were representative of previous studies.

Treatment with HA did not reduce joint swelling in the acute phase, as significant reduction of joint swelling was found only on day 15 (i.e. in the early chronic phase of AIA). The temporary reduction of joint swelling may be a result of the reduced acute inflammation observed histologically at an earlier time point (day 8). This anti-inflammatory effect of HA is consistent with the effects previously reported in collagen-induced arthritis [22,40] and human RA [41,42].

Interestingly, however, while the joint swelling continued to progressively and spontaneously decrease in untreated AIA, it persisted in HA-treated animals after day 15, although the difference remained at the limits of significance (*P *= 0.06). The persistence of inflammation after prolonged application of HA (day 29) is further substantiated by significant proteoglycan loss in the epiphyseal growth plate, by significant persistence of both acute and chronic inflammation, and by significantly increased histological signs of articular cartilage damage. This biphasic course regarding joint inflammation and destruction after repeated application of high-molecular-weight HA has not been previously described. HA therefore probably shows only a limited temporal window of anti-inflammatory activity in arthritis.

Adverse reactions to HA have been described in human osteoarthritis, either after the first injection and subsequent intra-articular injections or at the beginning of a new treatment course. These adverse events consisted of pain and/or transient swelling of the injected joint, mostly mild or moderate in intensity [43-45]. The adverse reactions observed upon intra-articular treatment of human osteoarthritis are not comparable with the biphasic effects in the present study, since they were only short-lasting and limited to a period immediately following injection.

### Proteoglycans in the epiphyseal growth plate of the femoral condyle and the articular cartilage

AIA was accompanied by a significant loss of proteoglycans in the epiphyseal growth plate of untreated AIA rats in the short-term test (significantly ameliorated by HA; day 8). This proteoglycan loss is probably caused by the inflammatory micromilieu in the arthritic joint [46] and the adjacent periarticular bone marrow [47], in analogy to the alterations of the primary and secondary spongiosa in AIA [48]. The significant prevention of proteoglycan loss from the epiphyseal growth plate by HA at this time point may be due to the clear anti-arthritic effects of HA, as also documented by the significant decrease of acute inflammation on day 8 (Fig. 6a). Whether intra-articular treatment with HA indirectly influences the inflammatory changes in the bone marrow, thereby preventing proteoglycan loss in the epiphyseal growth plate, remains to be investigated.

Unexpectedly, repeated intra-articular HA treatment induced proteoglycan loss in the epiphyseal growth plate in the long-term test (day 29). This late loss of proteoglycans in the epiphysis under prolonged HA treatment suggests late proinflammatory effects of HA, as also indicated by significantly elevated levels of acute and chronic inflammation, as well as cartilage damage (Fig. 6).

Regarding the proteoglycan content within the articular cartilage, HA completely prevented the proteoglycan loss in the short-term test. This applied to the severe loss of proteoglycans in the superficial layer — the layer most strongly affected in the present study (37%) and also that most strongly affected in the fibronectin-mediated arthritis model [19]. This prevention also applied to deeper layers of the calcified cartilage, however, indicating either deep-reaching effects of intra-articular HA (up to 100 μm; see Fig. 1) or an indirect effect on the micromilieu in the epiphyseal bone core. Such profound chondroprotective effects of HA have not previously been reported in an *in vivo *arthritis model. Previous *in vitro *studies, in which the proteoglycan release from the cell matrix of bovine chondrocyte cultures was inhibited by HA, have suggested a covering of the matrix of as a mechanism for the chondroprotective capacity of HA [19].

### Acute and chronic inflammation, and cartilage damage

HA reduced the histological signs of acute inflammation in the short-term test (day 8). At this point, HA also showed a tendency (*P *= 0.06) to protect the joint again against cartilage damage (Fig. 6c). The tendency for decreased cartilage damage in the short-term test supports the assumption that HA forms a temporary protecting barrier over the cartilage, and thereby protects it against degradation [16,19,49]. The known reduction of free oxygen radicals [31], as well as the reduction of cytokines and other mediators of acute inflammation by HA [15,21], could contribute to both its chondroprotective capacity (Fig. 6c) and its anti-inflammatory effects (Fig. 6a) in early chronic AIA (day 8).

In the long-term test (day 29) HA treatment was associated with significantly higher histological signs of acute and chronic inflammation and, more importantly, with more severe cartilage damage. The late proinflammatory effects of repeated HA application, in parallel to the late loss of epiphysis proteoglycans and late damage of articular cartilage (see earlier), point to an interdependence of inflammation and damage in HA-treated AIA rats at this stage.

Significantly higher histological signs of articular cartilage damage after repeated HA application (day 29) are in contrast to a non-altered proteoglycan content of the articular cartilage at this time (Fig. 5). A seemingly normal proteoglycan content may therefore not be sufficient to exclude structural damage of the articular cartilage in arthritis. Consequently, more sensitive *in vivo *procedures will have to be established to reliably assess functional or structural cartilage alterations before irreversible damage occurs [50].

### Serum levels of cytokines and GAG

In the present study, the systemic levels of IL-6, TNF-α and GAG were not significantly influenced by intra-articular administration of high-molecular-weight HA, indicating that both anti-inflammatory effects (day 8) and proinflammatory effects (day 29) are locally restricted.

## Conclusion

In conclusion, HA appears to have a limited therapeutic window for local treatment of arthritis, as shown by amelioration of clinical signs (day 15), by prevention of proteoglycan loss in the articular cartilage and epiphyseal growth plate, and by prevention of structural cartilage damage, as well as by the reduction of acute inflammation in the arthritic joint (day 8).

Late aggravation of clinical signs (not significant, day 29), proteoglycan loss from the epiphyseal growth plate, and acute/chronic inflammation and structural cartilage damage at this time point strongly indicate biphasic effects of local HA treatment.

Whether these biphasic effects are due to accumulation of HA beyond pathological levels (which may be avoidable by single injection instead of repeated injection) or whether certain phases of the clinical course render the animals sensitive to the proinflammatory effects of HA remains the subject of future research, both in animal models and in human RA.

## Abbreviations

AIA = antigen-induced arthritis; ELISA = enzyme-linked immunosorbent assay; GAG = glycosaminoglycan; HA = hyaluronic acid; HL = HA-treated AIA rats, long-term test; HS = HA-treated AIA rats, short-term test; IL = interleukin; mBSA = methylated bovine serum albumin; PBS = phosphate-buffered saline; RA = rheumatoid arthritis; TNF-α = tumour necrosis factor alpha; UL = untreated AIA rats, long-term test; US = untreated AIA rats, short-term test.

## Competing interests

The co-authors J-H Ozegowski, P-J Müller, S Möller, G Peschel, A Roth, and RA Venbrocks published a patent for the use of sulfated hyaluronic acid for the prevention of inflammatory arthritis in 2000. This substance is not the same as that used for the experiments in the present study. (Ozegowski J-H, Müller P-J, Möller S, Peschel G, Roth A, Venbrocks RA: Pharmazeutische Formulierungen zur Hemmung von entzündlichen Arthritiden [Use of hyaluronic acid derivatives for the prevention of inflammatory arthritis], HA 00-52 2000).

A Roth has to publish papers in peer-reviewed journals as a part of the process to finish his thesis (habilitation in Germany). This is a non-financial academic interest.

## Authors' contributions

AR carried out all experiments, the measurements and evaluation of the data, as well as the statistics, and drafted, revised, finalised, and submitted the manuscript. JM developed the experimental basis for the measurements of proteoglycans in the serum and supervised the analysis, trained and supervised AR in the analysis of proteoglycan loss from cartilage, actively participated in the analysis and evaluation of the data, and reviewed and contributed to the final version of the manuscript. AW participated in the preparation of the animals, fixation of the specimens, and blood collection, and reviewed the manuscript. RF actively participated in the experimental and organisational design of the study, gave valuable advice for the evaluation and interpretation of the experimental results, and reviewed the manuscript. AS reviewed all experimental data, gave valuable advice for the evaluation and interpretation of the experimental results and the subsequent conclusions, and reviewed the manuscript. RAV participated in the experimental and organisational design of the study, supervised the evaluation and interpretation of the experimental results, reviewed the manuscript, and organised financial support and laboratory space for the experiments. PP performed and summarised all histological analyses, and reviewed the manuscript. RB actively participated in the underlying animal experiments and the evaluation of the experimental results, and reviewed and contributed to the final version of the manuscript. HS actively participated in the underlying animal studies, provided and monitored all experimental animals, and reviewed the manuscript. JO, GP, and PJM developed the method for the production of HA, produced, purified, and quality-controlled the microbially manufactured, high-molecular-weight HA, and reviewed the manuscript. RWK participated in the experimental design of the study and the interpretation of the results, and reviewed and contributed to the initial and final version of the manuscript.
